# The *ColRS*-Regulated Membrane Protein Gene *XAC1347* Is Involved in Copper Homeostasis and *hrp* Gene Expression in *Xanthomonas citri* subsp. *citri*

**DOI:** 10.3389/fmicb.2018.01171

**Published:** 2018-06-11

**Authors:** Xiaojing Fan, Jing Guo, Yinghui Zhou, Tao Zhuo, Xun Hu, Huasong Zou

**Affiliations:** ^1^State Key Laboratory of Ecological Pest Control for Fujian and Taiwan Crops, Fujian University Key Laboratory for Plant-Microbe Interaction, Fujian Agriculture and Forestry University, Fuzhou, China; ^2^School of Agriculture and Biology, Shanghai Jiao Tong University, Shanghai, China

**Keywords:** *Xanthomonas citri* subsp. *citri*, *XAC1347*, membrane protein, *hrp* gene, regulation

## Abstract

*Xanthomonas citri* subsp. *citri* (*Xcc*) is the major causal agent of citrus canker disease. The *XAC1347* gene, which encodes a conserved membrane protein in *Xcc*, is required for virulence during infection. However, the molecular events mediated by *XAC1347* remain unclear. In this study, we reported that *XAC1347* gene is positively regulated by two component regulatory system *ColRS* and required for type III secretion system function. A non-polar deletion mutant of the *XAC1347* gene resulted in a Hrp minus phenotype in plants and reduced copper homeostasis. Real-time PCR experiments indicated that *XAC1347* gene is induced by copper ions. The expression levels of representative genes from four *hrp* operons, including *hrpB1, hrcV, hrpF*, and *hrpD6*, were reduced in *XAC1347* mutant, indicating that *XAC1347* is involved *hrp* gene expression.

## Introduction

*Xanthomonas citri* subsp. *citri* (syn. *Xanthomonas axonopodis* pv. *citri*; *Xcc*) is a causal agent of canker disease in citrus plants (Vauterin et al., 1995). Citrus canker is a serious disease which causes great concern to citrus producers worldwide. *Xcc* is naturally spread by rain splash, invades host plants through natural openings, and multiplies in the intercellular spaces (Graham et al., 1992). The typical symptoms of citrus canker include necrotic lesions surrounded by oily, water-soaked margins, and yellow chlorotic rings (Brunings and Gabriel, 2003).

The two-component system *colRS* was originally identified from *Pseudomonas fluorescens* strain WCS365, and was shown to play a critical role in root-colonization (Dekkers et al., 1998). Subsequently, it was found to be involved in the regulation of membrane function (Hõrak et al., 2004; de Weert et al., 2006; Kivistik et al., 2006; Yan and Wang, 2011; Mumm et al., 2016), transposition of a transposon (Kivistik et al., 2006), and phenol tolerance (Kivistik et al., 2006). Its role in tolerance to excess iron stress has been extensively studied by addition of zinc, manganese, or cadmium into culturing medium (Hu and Zhao, 2007; Ainsaar et al., 2014). In *X. oryzae* pv. *oryzae, colRS* mutants exhibit a decrease in virulence, a inability to grow on low-iron media and inability to induced HR on non-host tomato (Subramoni et al., 2012). The *colRS* system of *Xcc* exerts multiple regulatory roles in biological processes, including *in planta* growth, biofilm formation, catalase activity, lipopolysaccharide production, and resistance to environmental stress (Yan and Wang, 2011). Mutation in this system results in reduced tolerance to copper iron stress.

The type III secretion system (T3SS) is essential for pathogenic bacteria to deliver type III effectors into plant cells, and in determining effector-triggered susceptibility (ETS) or effector-triggered immunity (ETI) (Alfano and Collmer, 1997). In *Xcc*, T3SS is encoded by a cluster of HR and pathogenicity (*hrp*) genes organized into six *hrp* operons (Dunger et al., 2005). The *hrp* genes located in the *hrpB, hrpD*, and *hrpF* operons are essential for pathogenicity in citrus and the HR in non-host plants (Dunger et al., 2005). In addition to the key transcriptional activator *hrpX* (Guo et al., 2011), the *ColRS* system is also required for the optimal expression of *hrpD6* and *hpaF* genes encoding components of T3SS (Yan and Wang, 2011). The *hrpX* is located outside of the *hrp* gene cluster, while *hrpD6* in the *hrpD* operon has been reported to have a regulatory role on *hpa2, hpa1, hpaB, hrcC*, and *hrcT* in *X. oryzae* pv. *oryzicola*, which causes a bacterial stripe disease in rice plants (Li et al., 2011b).

Membrane bound proteins are the fundamental structure of bacterial cell membranes involved in cell signaling, adhesion and transportation (Vinothkumar and Henderson, 2010). To build a functional T3SS, HrcQ, HrcR, HrcS, HrcT, HrcU, and HrcV are localized to integral inner membrane proteins to form the inner core of the T3SS, while HrcJ and HrcC are localized to membrane periplasm (Deng and Huang, 1999; Tampakaki et al., 2004; Berger et al., 2010). An *Xcc* periplasmic protein VrpA physically interacts with T3SS components HrcJ and HrcC to mediate efficient effector protein secretion (Zhou et al., 2015). The outer membrane protein OprB is more abundant in *Xcc* biofilm. An *oprB* mutant is impaired in biofilm formation, adherence to the host, and virulence (Ficarra et al., 2017). Through proteomics and transposon-based analysis, a large number of outer membrane proteins, transporters and membrane-bound receptors have been identified to be associated with *Xcc* infection (Yan and Wang, 2012; Facincani et al., 2014; Song et al., 2015; Ferreira et al., 2016; Carnielli et al., 2017).

The contribution of the outer membrane protein XAC1347 to canker development was previously discovered by random Tn5 mutagenesis of *Xcc* (Yan and Wang, 2012; Song et al., 2015). Tn5 insertion in *XAC1347* gene did not affect *Xcc* growth on minimal medium but led to a remarkable reduction in canker symptom (Yan and Wang, 2012), and two *XAC1347* insertion mutants of strain *Xcc* 29-1 were impaired in pathogenicity (Song et al., 2015). In this study, a non-polar deletion mutant of *XAC1347* was constructed to determine how XAC1347 affected bacterial virulence and whether it was involved in environmental adaptation.

## Materials and Methods

### Bacterial Strains, Plasmids, and Culture Conditions

Bacterial strains and plasmids used in this study are listed in **Supplementary Table S1**. The *Xcc* strains were cultivated in nutrient broth (NB) medium or NB added with 1.5 % agar (NA) at 28°C (Song et al., 2015). To study protein secretion, western blotting was performed by culturing *Xcc* in XVM2, which mimics a plant-like environment (Guo et al., 2011). The minimal medium M9 was used to test whether the mutants were autotrophic. *Escherichia coli* strains were routinely cultured in Luria–Bertani medium at 37°C. *Agrobacterium tumefaciens* GV3101 was cultured in yeast extract and beef extract medium at 28°C. Antibiotics were applied at the following concentrations: kanamycin (Km) at 50 μg/ml, rifampin (Rif) at 50 μg/ml, spectinomycin (Sp) at 50 μg/ml, and gentamycin (Gm) at 10 μg/ml.

### Mutant Generation

The non-polar mutant of *ΔXAC1347* was constructed using homologous recombination in the *Xcc* 29-1 genetic background (Gicharu et al., 2016). The flanking DNA fragments were PCR amplified from *Xcc* 29-1 genomic DNA using the primer pairs 1347.1.F/1347.1.R and 1347.2.F/1347.2.R (**Supplementary Table S2**). These two flanking fragments were ligated into the vector pKMS1 at the *Xba*I and *Pst*I sites, resulting in pKMS-1347 (**Supplementary Table S1**). The recombinant plasmid was introduced into wild-type *Xcc* 29-1 for deletion mutant isolation (Gicharu et al., 2016). The primer sets 1347.1.F/1347.2.R was used to identify the desired deletion mutants *ΔXAC1347*. The deletion of targeted fragments was confirmed by sequencing the PCR products obtained from each mutant.

A site-directed mutagenesis was employed to construct the response regulator *ColR* insertion mutant. The 399-bp DNA fragment of partial *ColR* gene was PCR amplified and cloned into suicide vector pK18mob at *Eco*RI and *Hin*dIII, generating pK18-ColR. The resulted construct was introduced into wild type *Xcc* 29-1 to generate the insertion mutant *ΔColR* (Schäfer et al., 1994).

### Construction of Complementary Plasmids

The primer pairs C1347.F/C1347.R and CColR.F/CColR.R were used to PCR amplify *XAC1347* and *ColR* with their promoter region, respectively (**Supplementary Table S2**). The PCR products were separately inserted into pBBR1MCS-5, generating pBB-1347 and pBB-ColR. Each complementary plasmid was transformed into corresponding mutant for phenotype restoration analysis. The pBB-1347 was additionally transformed into a Tn5 insertion mutant Mxac111-54 to make comparison with XAC1347 deletion mutant. The mutant Mxac111-54 carried a Tn5 insertion site at position 40 from translation start codon in XAC1347, and showed a loss of pathogenicity on citrus plants (Song et al., 2015).

### Analysis of Bacterial Homeostasis to Copper

*Xcc* 29-1, *ΔXAC1347*, Tn5 insertion mutant Mxac111-54 and the corresponding complementation strains were cultured in NB broth and grown at 28°C for 24–36 h until the OD_600_ = 0.8. The cells were sub-cultured (1:100) in 4 ml fresh NB and incubated for another 16 h until the OD_600_ reached 0.6. After centrifugation at 6,000 rpm for 10 min at 4°C, the cell pellets were washed twice with sterilized water, and then re-suspended with sterilized water to an OD_600_ = 1.0. Cell suspensions were sub-cultured (1:100) in NB supplemented with gradient concentrations of copper ion (0.1, 0.2, 0.3, 0.4, and 0.5 mM) generated by adding copper sulfate to media. Growth rates were assessed by measuring OD_600_ values every 4 h over 2 days after sub-culturing. All of the experiments were repeated at least three times.

### Pathogenicity and HR Assays

The cultured *Xcc* strains were suspended in sterile distilled water to a final concentration of 10^8^ colony forming units (CFU)/ml (OD_600_ = 0.3). For the pathogenicity assay, bacterial suspensions were injected into fully expanded grapefruit (*Citrus paradise* Macf. cv Duncan) leaves with a needleless syringe. Disease symptoms were scored and photographed 5 days post inoculation. For the HR assay, bacteria were infiltrated into *Lycopersicon esculentum* leaves. Plant reactions were viewed 2 days post inoculation. Each test was repeated at least three times.

For the growth assay *in planta*, 0.8-cm-diameter leaf disks were cut with a cork borer. After surface sterilization twice with 75% ethanol, the disks were ground completely in 1 ml of sterile double distilled water. Serial dilutions of the suspension were plated on NA plates supplemented with appropriate antibiotics. Plates were incubated at 28°C for 3–4 days and the individual colonies were counted to determine the approximate CFU/cm^2^ of leaf area. The standard deviation was calculated based on the colony counts for three triplicate disks taken from each of the three samples per time point per inoculum. Experiments were repeated three times.

### Western Blot

To determine whether XAC1347 protein was secreted by *Xcc*, c-Myc-tagged XAC1347 was expressed in the broad host vector pBBR1MCS-5. A fused c-Myc-tag was introduced at the 3′-terminal in the reverse primer 1347.S.R (**Supplementary Table S2**). The 670-bp fused DNA fragment containing the full-length *XAC1347* gene was cloned into pBBR1MCS-5 at the *Xba*I and *Sac*I sites. The resulting recombinant plasmid pBB-1347S was introduced into wild-type *Xcc* 29-1. The *Xcc* 29-1(1347S) was cultured in NB medium to the logarithmic phase. Bacterial cells were harvested and re-suspended in fresh NB (OD_600_ = 0.2) or XVM2 (OD_600_ = 1.0) medium. After suspension, cells were cultured at 28°C for 16 h, bacterial cells and the corresponding supernatant fractions were separated by centrifugation, and the protein in the supernatant fraction was precipitated with 12.5% trichloroacetic acid (Li et al., 2011a). Proteins were separated on 10% SDS-PAGE gels and were transferred to membranes for immunoblotting using anti-c-Myc primary antibodies (HuaAn Biotechnology, Hangzhou, China). Primary antibodies were recognized by anti-rabbit secondary antibodies (HuaAn Biotechnology) and were visualized on autoradiographs using the Western-Light chemiluminescence system (Transgene, Beijing, China).

### GUS Activity Assay

To construct *XAC1347* promoter–GUS fusion, the promoter region upstream of the *XAC1347* open reading frame was PCR amplified from genomic DNA using primers 1347.P.F and 1347.P.R (**Supplementary Table S2**). The 542-bp PCR product was fused with the *gusA* gene in pRG960 vector at *Pst*I-*Bam*HI sites (**Supplementary Table S2**). The resulting construct was introduced into wild-type *Xcc* 29-1, *ColR* mutant *ΔColR*, and complementary strain C*ΔColR*, and the transformed strains were cultured in NB until the OD_600_ reached 0.8. After centrifugation at 6,000 rpm for 10 min at 4°C, the cell pellets were re-suspended in NB to OD_600_ = 1.0. Copper sulfate was added to cell suspension to produce a final copper concentration of 0.3 mM. After the cultures were cultivated for 4 h, cells were collected for GUS activity analysis (Li et al., 2011b). The GUS activities were measured using the GloMax Multi Detection System (Promega, Madison, WI, United States) with *p*-nitrophenyl-D-glucuronide as the substrate in a 1-h reaction. The GUS activity for each sample was read, and empty NB media were used as blank. The relative GUS activity was calculated as the GUS activities from *ΔColR* and C*ΔColR* divided by that from wild type 29-1. Assays were independently repeated three times.

### RNA Isolation and qRT-PCR

To evaluate gene expression levels in the bacteria cultured in liquid media, RNA was extracted from *Xcc* cells using the RNAprep pure Kit for Cell/Bacteria (Tiangen Biotech, Beijing, China). To study the expression of *XAC1347* under copper stress condition, the *Xcc* cells were cultured in NB medium till OD_600_ = 1.5, and then copper sulfate was supplemented and co-cultured for 4 h. To obtain RNA from cells growing in citrus, *Xcc* strains were infiltrated into fully expanded citrus leaves. At 24 h post inoculation, leaves were collected for RNA extraction using the Plant RNA Kit (Omega, Norcross, GA, United States) to isolate RNAs from grapefruit leaves. The total RNAs were quantified by measuring the OD_260_/OD_280_ ratio and RNA quality was analyzed using gel electrophoresis. Genomic DNA contamination was removed using the PrimeScript RT reagent Kit with gDNA Eraser (perfect Real Time) (Takara, Dalian, China) before reverse transcription. All of the primers used for qRT-PCR are listed in **Supplementary Table S3**. Assays were performed using the Applied Biosystems 7500 real-time PCR system with SYBR Premix Ex Taq (Takara). The PCR thermal cycle conditions were as follows: denaturation at 95°C for 30 s and 40 cycles of 95°C for 5 s and 60°C for 30 s. The expression of *gyrA* was used as the internal control.

### Transient Expression in Tobacco Leaves

To investigate the membrane bound trait of *XAC1347*, the *XAC1347* open reading frame was inserted into the pGDG vector (Goodin et al., 2002), generating an N-terminal fusion with the GFP gene under the control of the double cauliflower mosaic virus 35S promoter (**Supplementary Table S2**). The resulting pGDG-1347 and the pGDG empty vector control were transformed into *A. tumefaciens* strain GV3101. The infiltration manipulation was performed as described previously (Li et al., 2011b). Two days after inoculation, samples were imaged under a confocal laser scanning microscope (CLSM, Leica TCS SP5 II, Germany). Experiments were independently repeated three times.

## Results

### XAC1347 Is Alanine-Rich and Conserved in Plant Pathogenic *Xanthomonas*

**FIGURE 1:**
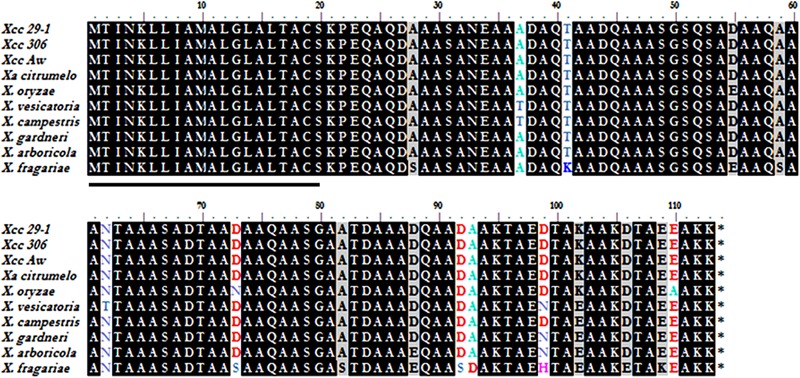
Sequence analysis of XAC1347 protein. Amino acid sequence alignment of XAC1347 homologs from eight *Xanthomonas* species. The 20 amino acids of signal peptide are underlined at N-terminus. Proteins involved are listed as follows: *Xcc* 29-1 (*X. citri* subsp. *citri* 29-1, AGH76857.1), *Xcc* 306 (*X. citri* subsp. *citri* 306, AAM36218.1), *Xcc* A^w^ (*X. citri* subsp. *citri* A^w^, AGI08775.1), *Xa citumelo* (*X. axonopodis* pv. *citrumelo* F1, AEO41615.1), *X. oryzae* (WP_019302275.1), *X. vesicatoria* (WP_005992008.1), *X. campestris* (NP_636670.1), *X. gardneri* (WP_006449860.1), *X. arboricola* (WP_016904096.1), and *X. fragariae* (WP_002810337.1).

*XAC1347* is a 339-bp-long gene coding for a 113-amino acid small protein, which does not contain phenylalanine, histidine, valine, tyrosine, or tryptophan, but contains 49 alanines, accounting for approximately 43.3% of the total amino acids. Particularly, there are six –AAA- and nine –AA- repeats in this small protein (**Figure 1**). The first 20 amino acids at the N-terminus comprises a putative a putative signal peptide, possibly required for translocation to cell membrane. The XAC1347 protein shows 100% identity among sequenced *Xcc* strains and one *X. axonopodis* pv. *citrumelo* F1 strain. Using multiple sequence alignment, nine amino acids are found different among homologs in *X. campestris, X. oryzae, X. vesicatoria, X. arboricola, X. fragariae*, and *X. gardneri*. The XAC1347 protein shows over 97% identity between those homologs (**Figure 1**).

### The Non-polar Deletion Mutant *ΔXAC1347* Was Impaired in Pathogenicity in Citrus and HR Induction in Tomato

**FIGURE 2:**
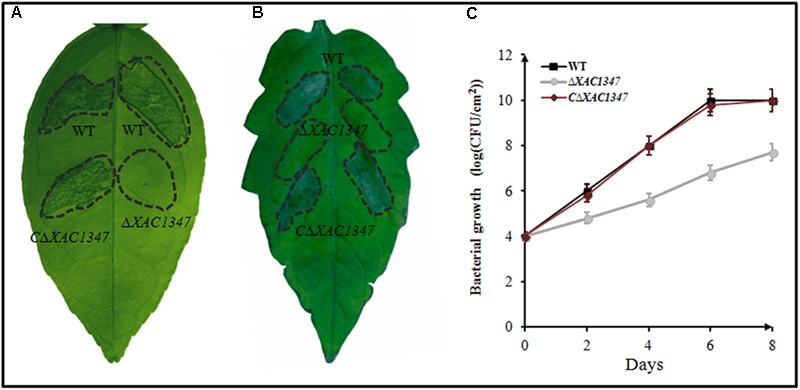
The phenotypes of non-polar deletion mutant *ΔXAC1347* in *Citrus paradise* and *Lycopersicon esculentum.* **(A)** Citrus canker on *C. paradise* Macf. cv Duncan. **(B)** The hypersensitive response on *L. esculentum.* **(C)** Bacterial growth in citrus plants. The bacterial cells were resuspended in sterile water at a concentration of 10^8^ CFU/ml and used to infiltrate cv. Duncan grapefruit leaves with a needleless syringe. The values shown are the means of three technical repeats with standard deviations.

A non-polar deletion mutant *ΔXAC1347* was produced by two steps of homologous recombination (**Supplementary Figure S1**). A 631-bp DNA fragment comprised of the *XAC1347* gene and its promoter was cloned into pBBR1MCS-5 to generate a recombinant pBB-1347 for complementation analysis. The wild-type strain 29-1, the deletion mutant *ΔXAC1347* and the complemented strain *CΔXAC1347* were individually infiltrated into the leaves of grapefruit and tomato. Similar to the phenotypes of Tn5 insertion mutants (Song et al., 2015), the deletion mutant *ΔXAC1347* produced no canker symptoms in citrus leaves (**Figure 2A**). The complemented *CΔXAC1347* strain restored the virulence in citrus to the level of that in the wild-type strain, producing typical canker symptoms (**Figure 2A**). At 48 h after inoculation into *L. esculentum* leaves, *ΔXAC1347* did not induce the HR, and the HR defect was restored by the *XAC1347* gene under the control of its own promoter (**Figure 2B**).

To evaluate the bacterial growth in host plants, the cell numbers were measured every 2 days after inoculation into citrus leaves. At 2 days after inoculation, the cell density of wild-type *Xcc* 29-1 reached 10^6^ CFU/cm^2^, exhibiting a high proliferation rate in the host plants. It reached 10^8^ CFU/cm^2^ at 4 days after inoculation, and reached 10^10^ CFU/cm^2^ at 6 days after inoculation. By contrast, the cell number of the deletion mutant *ΔXAC1347* was ∼10^5^ CFU/cm^2^ at 2 days after inoculation, nearly 10-fold lower than that of the wild type. At 4, 6, and 8 days after inoculation, the mutant’s growth rate remained slow, and the cell number was less than 10^8^ CFU/cm^2^ at 8 days after inoculation. The complementary strain *CΔXAC1347* restored the bacterial growth capability in citrus plants (**Figure 2C**).

### *XAC1347* Is Involved in the Expression of *hrp* Genes

**FIGURE 3:**
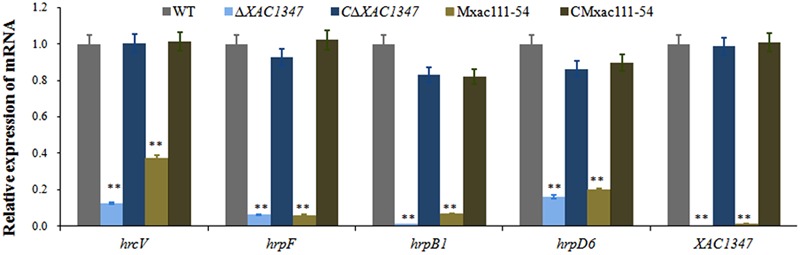
qRT-PCR analysis of *hrp* gene expression in *XAC1347* mutant. Total RNAs were extracted from *Xcc* cells growing in citrus at 24 h post inoculation, The *gyrA* was used as the internal control. Statistical analysis was conducted using Student’s *t*-test. Asterisks denote statistical significance as compared to wild type. ^∗∗^*P* < 0.01; *n* = 3.

Since the *XAC1347* mutant showed a typical T3SS defection phenotype, we therefore evaluate its effect on *hrp* gene expression. Because the *hrp* genes located in the *hrpB, hrpC, hrpE*, and *hrpF* operons are essential for pathogenicity in citrus (Dunger et al., 2005), four representative genes *hrpB1, hrcV, hrpF*, and *hrpD6* are chosen for further analysis. The representative *hrp* genes were all down regulated in *XAC1347* deletion mutant *ΔXAC1347* and Tn5 insertion mutant Mxac111-54. Specifically, the expression levels of *hrpB1* and *hrpF* were reduced over 90%, and the expression levels of *hrcV* and *hrpD6* were reduced over 85% (**Figure 3**). Our results indicate that *XAC1347* is required for the full expression of *hrp* genes.

### *XAC1347* Is Involved in Copper Homeostasis

**FIGURE 4:**
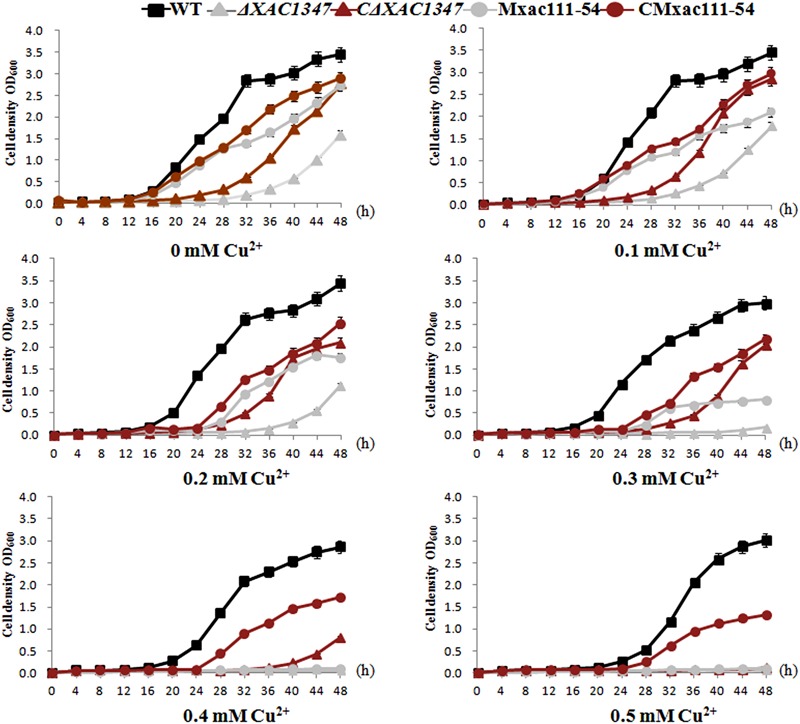
Growth curves of *XAC1347* mutants in response to copper stress. The cultured *Xcc* cells were adjusted to OD60 = 1.0 and sub-cultured (1:100) in NB medium supplemented with gradient concentrations of copper ions. Growth rates were assessed by measuring OD_600_ values every 4 h. All of the experiments were performed in triplicate and repeated three times with similar results. Error bars denote standard deviation of three experimental replicates.

XAC1347 is annotated as a membrane protein in the genome database (da Silva et al., 2002). We therefore tested whether it is involved in maintaining cell osmotic balance. Bacterial growth was assessed in nutrient NB liquid media by adding a gradation of copper sulfate concentrations, ranging from 0.1 to 0.5 mM. Wild-type *Xcc* 29-1 could grow in all five concentrations of copper ions, but with the increasing copper concentration, the growth rate declined (**Figure 4**). In contrast, the deletion mutant *ΔXAC1347* had reduced growth ability in 0.1 and 0.2 mM copper sulfate, and could not grow in media containing 0.3, 0.4, or 0.5 mM copper (**Figure 4**). The introduction of pBB-1347 into the mutant *ΔXAC1347* partially restored its homeostasis to copper. Similarly, the growth rate of Tn5 insertion mutant Mxac111-54 also declined as the copper concentration increased (**Figure 4**). Even though the copper homeostasis of the complementation strains was not restored to that of the wild type, the experiment indicated that *XAC1347* plays a role in copper homeostasis.

### The Transcription of *XAC1347* Gene Is Induced by Copper Ion

**FIGURE 5:**
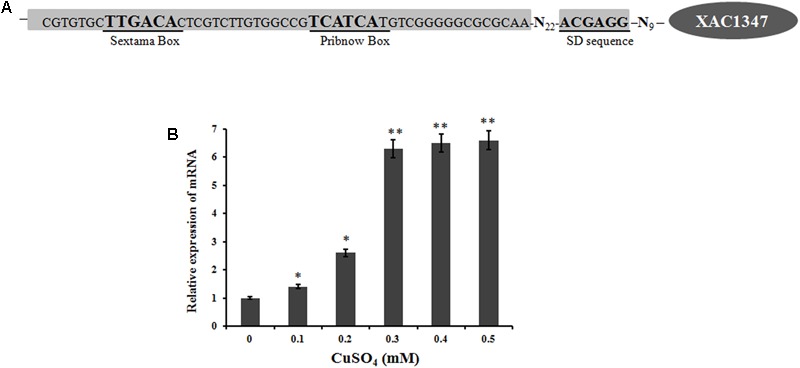
The transcription of *XAC1347* gene induced by copper. **(A)** Nucleotide sequence of *XAC1347* promoter region. Sextama box, Pribnow box, and SD sequence were underlined. **(B)** qRT-PCR analysis of *XAC1347* mRNA levels. The *gyrA* was used as the internal control. Statistical analysis was conducted using Student’s *t*-test. Asterisks denote statistical significance as compared to the control culturing in copper-free NB. ^∗^*P* < 0.05; ^∗∗^*P* < 0.01, *n* = 3.

The promoter region of *XAC1347* was predicted using the Berkeley Drosophila Genome Project^1^. A putative promoter with a 90% possibility score was predicted from 37 bp upstream of the transcriptional ATG start codon (**Figure 5A**). The promoter region also showed a high level of conservation in plant pathogenic *Xanthomonas* species, implying that its homologs may have similar expression patterns in other *Xanthomonas* strains.

A real-time qRT-PCR analysis was carried out to determine the mRNA level of *XAC1347* under copper-stress conditions. Based on a comparison with the expression level in copper-free NB medium, the *XAC1347* gene’s mRNA level was increased when copper sulfate was added. In NB media supplemented with 0.1 mM copper sulfate, the mRNA level increased 40%. The mRNA level was 2.6-fold in media supplemented with 0.2 mM copper sulfate relative to that in copper-free medium. At the 0.3, 0.4, and 0.5 mM concentrations of copper sulfate, the mRNA levels were increased by 6.3, 6.5, and 6.6-fold, respectively (**Figure 5B**). These data indicate that expression of *XAC1347* is induced by copper.

### *XAC1347* Gene Is Under Control of *ColRS* System

**FIGURE 6:**
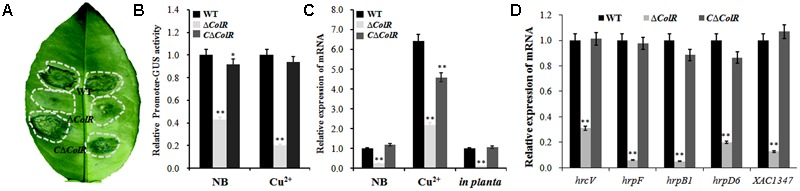
The transcriptions of *XAC1347* and *hrp* genes in *ColR* mutant. **(A)** Pathogenicity of *ColR* mutant in citrus plants. **(B)** *XAC1347* promoter activity monitored by β-glucuronidase (GUS). The relative GUS activities were calculated from the GUS activity of mutant divided by the GUS activity in wild type. **(C)** qRT-PCR analysis of *XAC1347* mRNA levels. **(D)** qRT-PCR analysis of *hrp* gene expressions. The *gyrA* was used as the internal control. (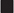), Wild type *Xcc* 291-1; (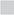) Mutant *ΔColR*; (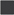) Complementary strain C*ΔColR*. Statistical analysis was conducted using Student’s *t*-test. Asterisks denote statistical significance as compared to the control culturing in copper-free NB. ^∗∗^*P* < 0.01, *n* = 3.

The two-component system *ColRS* plays important roles in several biological processes, including the pathogenicity and adaptation to copper stress (Yan and Wang, 2011). To determine whether *XAC1347* gene is regulated by *ColRS* system, a null mutant of response regulator *ColR* was constructed to mutagenize the *ColRS* system. The *ΔColR* did not produce canker symptom in citrus plants (**Figure 6A**). Next, *XAC1347* promoter-GUS fusion construct was introduced into *ΔColR* and its complementary strain C*ΔColR.* Compared with wild type, GUS activities were reduced in *ColR* mutant culturing either in NB medium or in media containing 0.3 mM copper sulfate (**Figure 6B**). Then, transcription level of *XAC1347* gene was assessed by qRT-PCR analysis. In **Figure 6C** transcription of *XAC1347* was reduced in *ΔColR* compared with that in wild type cultured in NB medium with or without copper. In citrus plants, the transcription of *XAC1347* gene showed similar pattern of reduced expression in *ColR* mutant (**Figure 6C**). Furthermore, the expression levels of the representative *hrp* genes were all reduced in the *ColR* mutant (**Figure 6D**). This indicated that *XAC1347* gene was positively regulated by *ColRS* in *Xcc.* We additionally assessed the *ColR* and *ColS* expression in *XAC1347* mutant *in planta*. Both genes did not show distinct expression level changes compared to expression in the wild type (**Supplementary Figure S2**).

## Discussion

The contribution of *XAC1347* to bacterial virulence has been reported in several previous publications (Yan and Wang, 2012; Song et al., 2015), but the molecular mechanisms have not been fully understood. In our previous work, two Tn5 insertion mutants of *XAC1347* were identified to have abolished virulence in citrus plants (Song et al., 2015). To gain a better understanding of this alanine-rich protein, a non-polar deletion mutant of *XAC1347* was constructed in this study. In addition to the loss of pathogenicity in citrus host and HR reaction in tomato, the mutant *ΔXAC1347* had reduced homeostasis to copper iron. The expression of *XAC1347* was copper-induced and controlled by the two-component system *ColRS*.

A previous study reported that an Tn5 insertion mutant of *XAC1347* gene derived from strain *Xcc* 306 did not show distinct growth alteration in minimal medium M9 (Yan and Wang, 2011). Our deletion mutant *ΔXAC1347* showed reduced growth rate in nutrient rich medium. To confirm phenotypic alteration in bacterial growth, the Tn5 insertion mutant Mxac111-54 was additionally studied. Like the deletion mutant *ΔXAC1347*, Mxac111-54 also reduced the growth in nutrient medium. In minimal medium M9, the colonies of *ΔXAC1347* and Mxac111-54 were smaller than those of wild type (**Supplementary Figure S3**). This demonstrated that mutation in XAC1347 did lead to a reduction in bacterial growth in our research. The inconsistent growth phenotype may have been due to that the mutants were constructed from different wild type *Xcc* strains.

The *ΔXAC1347* mutant did not induce canker symptom in citrus or HR reaction in tomato plant. This phenotype is resulted from the fact that the *hrp* genes were down regulated in *XAC1347* mutants. It has to be mentioned that the Hrp phenotype alteration is not caused by the slow growth, because 10^2^–10^3^ CFU/ml *Xcc* cells are sufficient to induce canker lesions through wounds (Goto, 1962; Zubrzycki and Diamante, 1987). In our study, the cell number of *ΔXAC1347* reached 10^5^ CFU/ml at 2 days post inoculation and 10^7^ CFU/ml at 6 days post inoculation in citrus tissue. Through promoter-GUS fusion and Real-time PCR experiments, *XAC1347* was found to be down regulated in *ColR* mutant cells cultured in NB medium, copper stress condition, or *in planta*. The *hrpC* and *hrpE* operons were positively regulated by the *ColRS* system in *X. campestris* pv. *campestris* and *X. oryzae* pv. *oryzicola* (Zhang et al., 2008; Li et al., 2011b). In *Xcc, ColRS* system controls multiple bacterial traits, like *hrp* gene expression, LPS production, catalase activity, and copper resistance (Yan and Wang, 2011). Four representative *hrp* genes, *hrcV, hrpD6, hrpF*, and *hrpB1*, which were all down-regulated in *XAC1347* mutant, were also regulated by *ColRS*. The collected data suggest that *XAC1347* is positively regulated by the *ColRS* system.

Proteins delivered by secretion systems in *Xcc* play important roles in canker development. The T3SS is responsible for the secretion of a repertoire of effector proteins, some of which are translocated directly into plant cells (Alfano and Collmer, 1997). There are at least 24 effector protein coding genes in the sequenced strain *Xcc* 29-1, including four *avr/pthA* genes, *pthA1, pthA2, pthA3*, and *pthA4* (Song et al., 2015). The *pthA4* homolog that carries a 17.5-tandem-repeat domain has been proposed as the main virulence determinant during infection (Song et al., 2015). Although the *XAC1347* mutant showed a Hrp deficiency similar phenotype, it remains unclear whether *XAC1347* is required for effector efficient secretion, functioning like the periplasmic protein VrpA (Zhou et al., 2015).

XAC1347 shows certain traits of a typical integral protein. Although it only contains 113-amino acids, the N-terminus 20 amino acids were predicted to form a signal peptide, which makes it possible that XAC1347 may function as an integral membrane protein (Wallin and von Heijne, 1998). By fusion with GFP, we did found that most of XAC1347 protein is bonded to cell membrane (**Supplementary Figure S4A**). As a biotrophic pathogen, *Xcc* penetrates the apoplast and releases a large number of molecules into the apoplastic space, some of which are pathogen-associated molecular patterns (PAMPs), such as lipopolysaccharides, flagellin or elongation factor Tu (Facincani et al., 2014; Ferreira et al., 2016; Carnielli et al., 2017). The western blot results indicated that the XAC1347-C-Myc protein was detected from the total cell extract, but not present in the culture supernatant (**Supplementary Figure S4B**). Thus, XAC1347 may not be secreted outside to plant apoplastic space during infection.

Because membrane integral proteins usually act as pathways for ions and molecules, we suspected that *XAC1347* might be involved in maintaining cell integrity and osmotic balance (Cybulski and de Mendoza, 2011; Kar et al., 2017). We therefore studied the role of *XAC1347* in copper homeostasis. Copper is an important transition metal for most organisms, but can be toxic at high levels. The mechanism for copper homeostasis has been studied in many prokaryotes. In *Pseudomonas syringae*, the plasmid-borne copper resistance operon *copABCD* is regulated by a copper-inducible promoter that is recognized by the regulatory genes *copR* and *copS* located downstream of *copD* (Cha and Cooksey, 1991; Mills et al., 1993). This suggests that bacteria employ a two-component system to mediate gene expression in response to copper stimuli and to regulate copper-resistance gene expression. Seven copper-resistance genes have been molecularly identified from *Xcc* A44; however, only three genes (*copL, copA*, and *copB*) were found in the sequenced strains *Xcc* 306 and *Xcc* 29-1 (Teixeira et al., 2008; Behlau et al., 2011). Additionally, *copR*/*copS* were not found in all *Xcc* strains (da Silva et al., 2002; Behlau et al., 2011). It is still unclear which member of the two-component system is responsible for regulating copper homeostasis in *Xcc*. Even though mutations in *ColRS* reduced bacterial tolerance to copper, no evidence has been presented if *copLAB* was directly regulated by *ColRS* (Yan and Wang, 2011). In this study we found that the transcription of *XAC1347* was increased under copper stress and *XAC1347* was essential for copper homeostasis in *Xcc*. Further studies are needed to elucidate the exact mechanism employed by *XAC1347* to mediate bacterial copper homeostasis.

## Conclusion

XAC1347 in *Xanthomonas citri* subsp. *citri* is alanine-rich membrane protein involved in copper homeostasis. The transcription of *XAC1347* gene is induced by copper iron and under the control of two component regulatory system *ColRS*. Mutation in *XAC1347* resulted in significantly reduced expression of *hrp* genes, which may be the reason why the *ΔXAC1347* mutant fails to cause canker symptom in citrus.

## Author Contributions

XF and JG performed the research. YZ, TZ, and XH analyzed the data. HZ designed the research and wrote the manuscript.

## Conflict of Interest Statement

The authors declare that the research was conducted in the absence of any commercial or financial relationships that could be construed as a potential conflict of interest.

## Footnotes

1 http://www.fruitfly.org/seq_tools/promoter.html

## Supplementary Material

FIGURE S1 Molecular analysis of the *ΔXAC1347* mutant of *Xanthomonas citri* subsp. *citri.* **(A)** PCR analysis of the *ΔXAC1347* mutant. The size difference of PCR products from wild-type *Xcc* 29-1 and *ΔXAC1347* was revealed using primers 1347.1.F and 1347.2.R. **(B)** Southern blot of the *ΔXAC1347* mutant. The Southern blot was carried out using the 342-bp *XAC1347* gene as the probe against genomic DNA digested with *Not*I.

FIGURE S2 qRT-PCR analysis of the transcription of *ColR* and *ColS* in *XAC1347* mutant. The *gyrA* was used as the internal control. Statistical analysis was conducted using Student’s *t*-test. Asterisks denote statistical significance as compared to wild type. ^∗^*P* < 0.05; ^∗∗^*P* < 0.01, *n* = 3.

FIGURE S3 XAC1347 mutants grown on solid minimal medium M9. The cultured *Xcc* cells were adjusted to OD60 = 1.0 in liquid M9 medium, and then serially diluted by 10-fold to make further concentrations of OD_600_ = 0.1, 0.01, 0.001. For each series, 2 μl cell suspension was dropped on M9 plates. Cell colonies were viewed at 6 days post inoculation

FIGURE S4 Membrane bound trait and secretion analysis of XAC1347 protein. **(A)** Subcellular localization of *XAC1347* in *Nicotiana benthamiana*. Bars, 15 μm; LM, light microscopy; FM, fluorescence microscopy, Mer, Merge. **(B)** The secretion of XAC1347 in *Xanthomonas citri* spp. *citri*. A c-Myc-tagged XAC1347 was expressed under the control of its own promoter. The cell pellets were ultrasonicated for total extraction (TE) and the protein in the supernatant fraction (SN) was precipitated with 12.5% trichloroacetic acid.

TABLE S1 Bacterial strains and plasmids used in this study.

TABLE S2 Primers for molecular cloning in this study.

TABLE S3 Primers for real time PCR analysis in this study.
