# Supplementary figures and images for: The ColRS-Regulated Membrane Protein Gene XAC1347 Is Involved in Copper Homeostasis and hrp Gene Expression in Xanthomonas citri subsp. citri
Source: Front Microbiol. 2018 Jun 11;9:1171. doi: 10.3389/fmicb.2018.01171 (PMC6004745; doi:10.3389/fmicb.2018.01171)

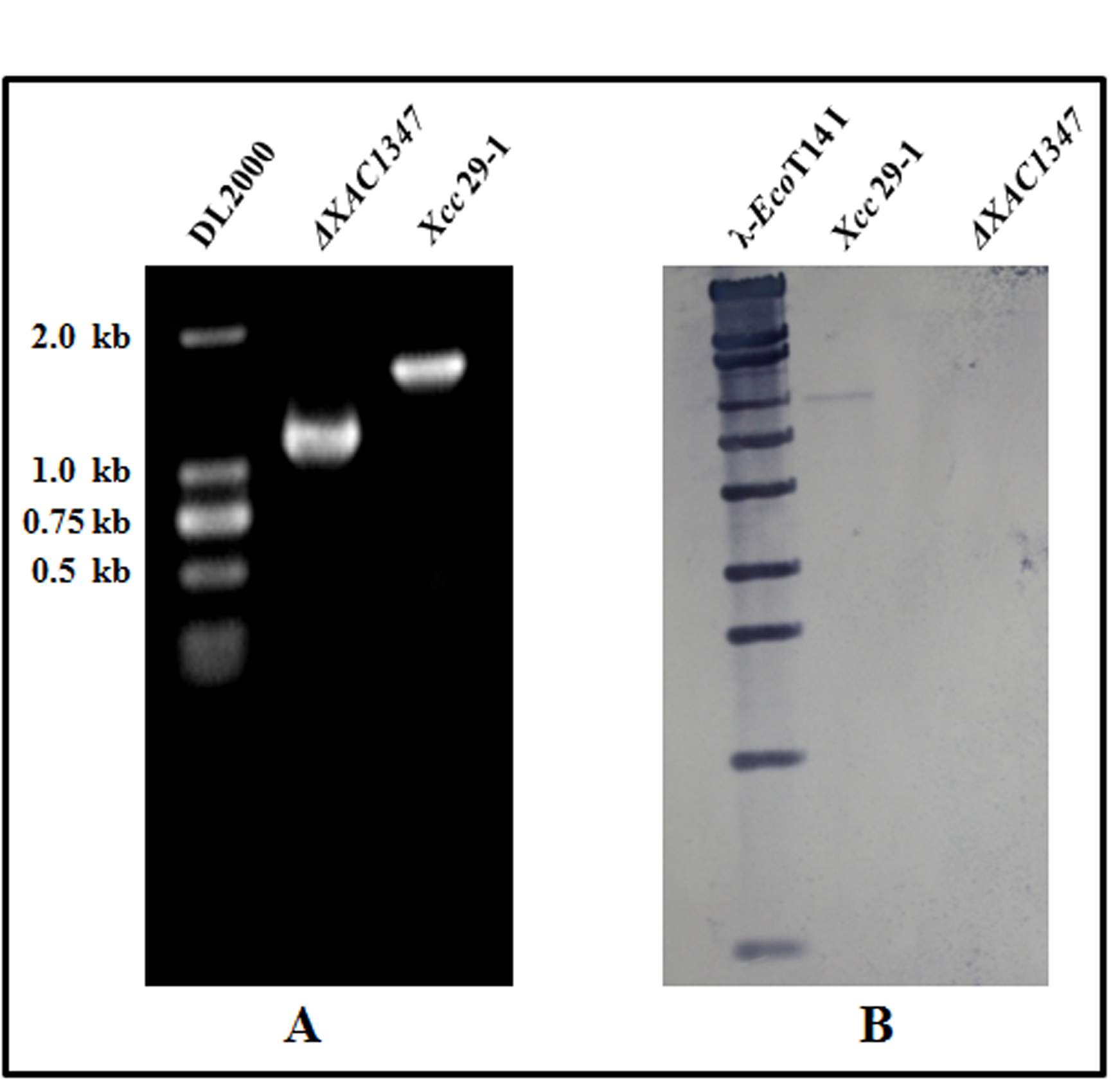

Supplement: FIGURE S1 — Molecular analysis of the ΔXAC1347 mutant of Xanthomonas citri subsp. citri. (A) PCR analysis of the ΔXAC1347 mutant. The size difference of PCR products from wild-type Xcc 29-1 and ΔXAC1347 was revealed using primers 1347.1.F and 1347.2.R. (B) Southern blot of the ΔXAC1347 mutant. The Southern blot was carried out using the 342-bp XAC1347 gene as the probe against genomic DNA digested with NotI. [file Image_1.TIF]

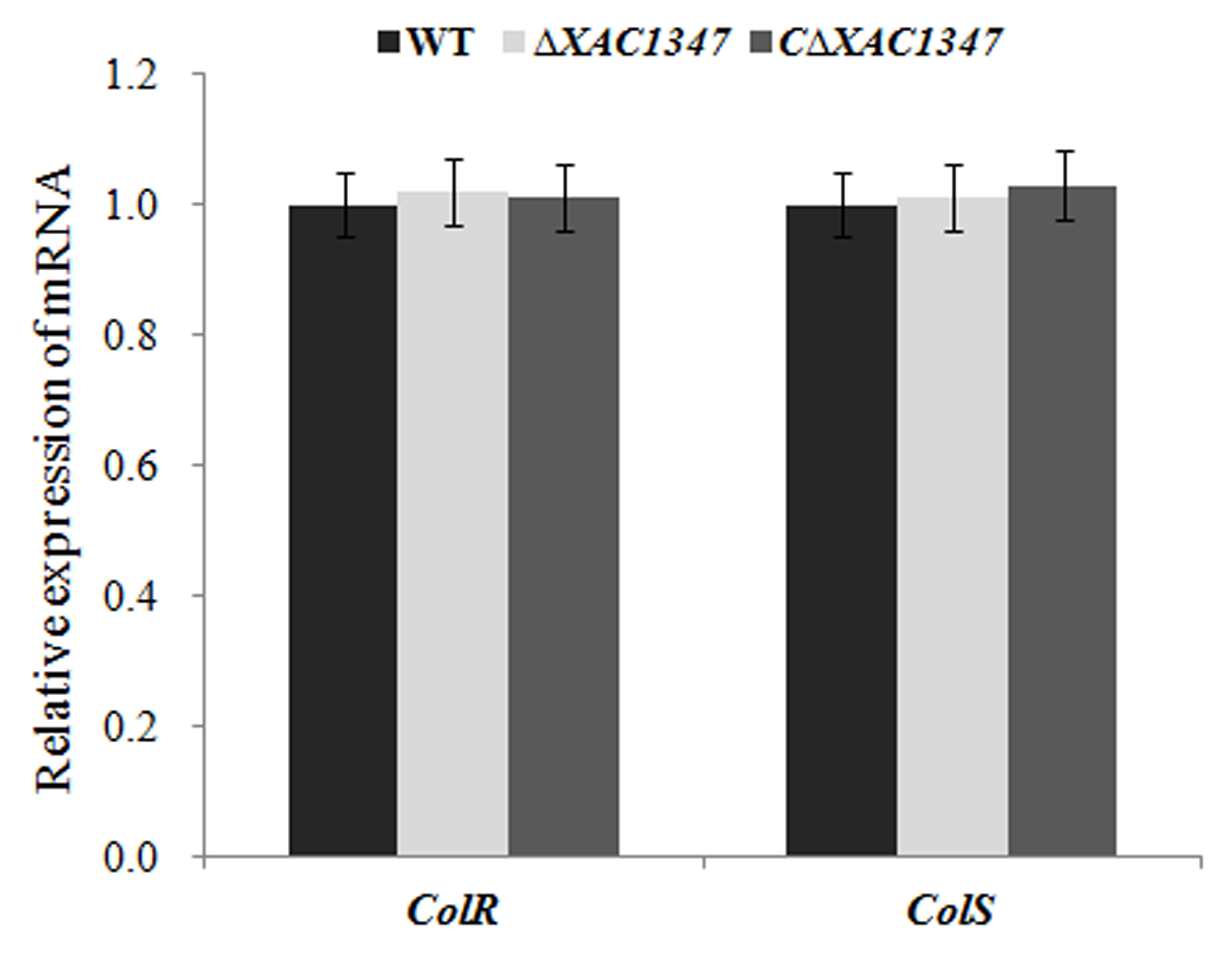

Supplement: FIGURE S2 — qRT-PCR analysis of the transcription of ColR and ColS in XAC1347 mutant. The gyrA was used as the internal control. Statistical analysis was conducted using Student’s t-test. Asterisks denote statistical significance as compared to wild type. ∗P < 0.05; ∗∗P < 0.01, n = 3. [file Image_2.TIF]

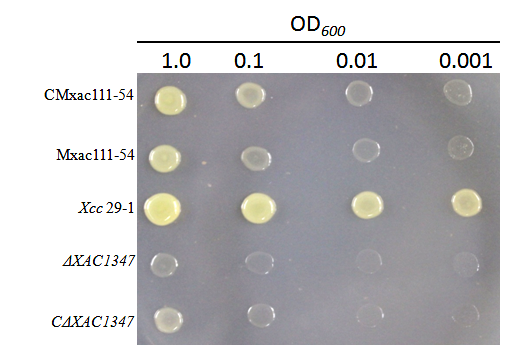

Supplement: FIGURE S3 — XAC1347 mutants grown on solid minimal medium M9. The cultured Xcc cells were adjusted to OD60 = 1.0 in liquid M9 medium, and then serially diluted by 10-fold to make further concentrations of OD600 = 0.1, 0.01, 0.001. For each series, 2 μl cell suspension was dropped on M9 plates. Cell colonies were viewed at 6 days post inoculation [file Image_3.TIF]

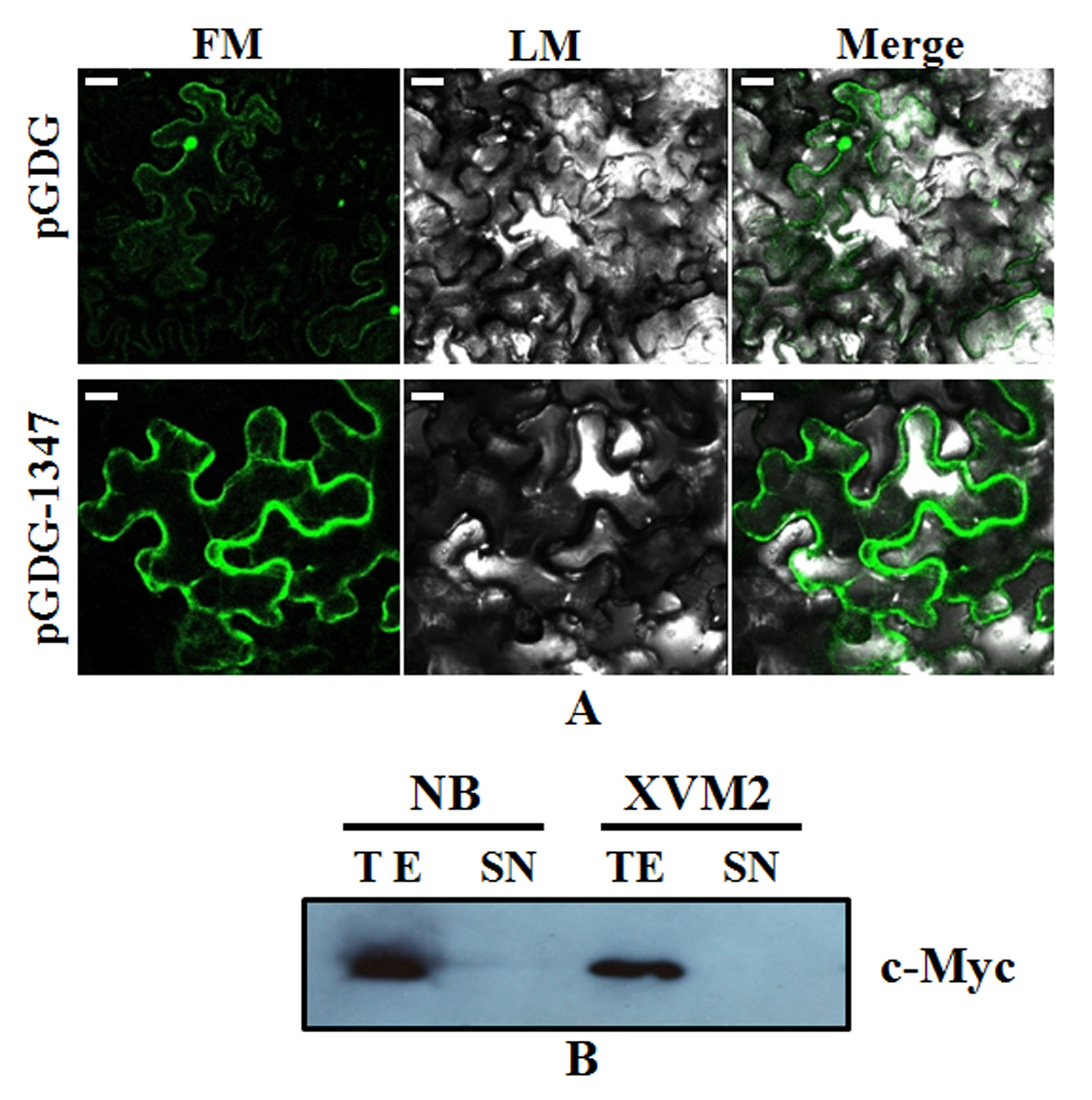

Supplement: FIGURE S4 — Membrane bound trait and secretion analysis of XAC1347 protein. (A) Subcellular localization of XAC1347 in Nicotiana benthamiana. Bars, 15 μm; LM, light microscopy; FM, fluorescence microscopy, Mer, Merge. (B) The secretion of XAC1347 in Xanthomonas citri spp. citri. A c-Myc-tagged XAC1347 was expressed under the control of its own promoter. The cell pellets were ultrasonicated for total extraction (TE) and the protein in the supernatant fraction (SN) was precipitated with 12.5% trichloroacetic acid. [file Image_4.TIF]
