# Supplementary material for: The ColRS-Regulated Membrane Protein Gene XAC1347 Is Involved in Copper Homeostasis and hrp Gene Expression in Xanthomonas citri subsp. citri
Source: Front Microbiol. 2018 Jun 11;9:1171. doi: 10.3389/fmicb.2018.01171 (PMC6004745; doi:10.3389/fmicb.2018.01171)
Supplement: TABLE S1 — Bacterial strains and plasmids used in this study. [file Table_1.DOCX]

Table S1. Bacterial strains and plasmids used in this study

| **Strains or plasmids** | **Relevant characteristics** | **Resources** |
| --- | --- | --- |
| **Strains** | | |
| *Xanthomonas citri* subsp. *citri* | | |
| *Xcc* 29-1 | Wild-type | This lab |
| *ΔXAC1347* | A non-polar mutant of *XAC1347* derived from *Xcc* 29-1 | This study |
| *CΔXAC1347* | Gm^r^, *ΔXAC1347* carrying pBB-1347 | This study |
| *ΔColR* | Km^r^, a insertion mutant of *ColR* gene derived from *Xcc* 29-1 | This study |
| *CΔColR* | Gm^r^, *ΔColR* carrying pBB-ColR | This study |
| *ΔColR*(P1347GUS) | *ΔColR* carrying pRG960-P1347 | This study |
| *CΔColR* (P1347GUS) | *CΔColR* carrying pRG960-P1347 | This study |
| *Xcc* 29-1(1347S) | Gm^r^, *Xcc* 29-1 carrying pBB-1347S | This study |
| *Xcc* 29-1(P1347GUS) | Km^r^, *Xcc* 29-1 carrying pRG960-P1347 | This study |
| *Agrobacterium tumefaciens* | | |
| GV3101 | Rif^r^, with Ti plasmid pMP90 | Koncz and Schell, 1996 |
| GV3101(GFP) | Rif^r^, Km^r^,GV3101 carrying pGDY empty vector | This study |
| GV3101(1347GFP) | Rif^r^, Km^r^,GV3101 carrying pGDY-*XAC1347* | This study |
| *Escherichia coli* | | |
| DH5α | *F- recA hsdR17 (rk−, mk+) ϕ80lacZ∆M15* | Clontech |
| **Plasmids** |  |  |
| pKMS1 | Km^r^, suicide vector derivative from pK18mobGII, *sacB*^+^ | This lab |
| pKMS-1347 | Km^r^, a 1247 bp fusion cloned in pKMS1 for *XAC1347* deletion construction | This study |
| pK18mob | Km^r^, suicide vector | Schäfer et al., 1994 |
| pK18-ColR | Km^r^, a 399-bp *ColR* gene coding sequence inserted in pK18mob | This study |
| pBBR1MCS-5 | Gm^r^, 4.7-kb broad-host range plasmid, *lacZ* | Kovach *et al*., 1994 |
| pBB-1347 | Gm^r^, a 631bp DNA fragment containing *XAC1347* and its promoter cloned in pBBR1MCS-5 | This study |
| pBB-1347S | Gm^r^, a 670 bp DNA fragment expressing C-Myc tagged XAC1347 fusion in pBBR1MCS-5 | This study |
| pBB-ColR | Gm^r^, a 631bp DNA fragment containing *ColR* and its promoter cloned in pBBR1MCS-5 | This study |
| pGDG | Km^r^, GFP transient expression vector | Goodin *et al*., 2002 |
| pGDG-1347 | Kmr, a 342 bp XAC1347 gene in pGDY for expressing XAC1347-GFP fusion | This study |
| pRG960 | Sp^r^, Broad-host-range vector carrying a promoterless *gusA* gene with start codon | Van den Eede et al. 1992 |
| pRG960-P1347 | Km^r^, derivated from pCAMBIA1301 which encodes the C-terminal portion of YFP | This study |

**REFERENCE**

De Feyter, R., Kado, C. I. and Gabriel, D. W. (1990). Small, stable shuttle vectors for use in *Xanthomonas*. *Gene* 88, 65–72.

Goodin, M. M., Dietzgen, R. G., Schichnes, D., Ruzin, S., and Jackson, A. O. (2002).pGD vectors: versatile tools for the expression of green and red fluorescent protein fusions in agroinfiltrated plant leaves. *Plant J.* 31, 375–383.

Koncz, C., and Schell, J. (1986). The promoter of TL-DNA gene 5 controls the tissuespecific expression of chimeric genes carried by a novel type of *Agrobacterium* binary vector. *Mol. Gen. Genet.* 204, 383–396.

Kovach, M. E., Phillips, R. W., Elzer, P.H., Roop, R., and Peterson, K. M. (1994). pBBR1MCS: a broad-host-range cloning vector. *Gene* 166, 175-176.

Van den Eede, G., Deblaere, R., Goethals, K., Van Montagu, M., and Holsters, M. (1992). Broad host range and promoter selection vectors for bacteria that interact with plants. *Mol. Plant-Microbe Interact.* 5, 228-234.

Schäfer, A., Tauch, A., Jäger, W., Kalinowski, J., Thierbach, G., and Pühler, A. (1994). Small mobilizable multi-purpose cloning vectors derived from the *Escherichia coli* plasmids pK18 and pK19: selection of defined deletions in the chromosome of *Corynebacterium glutamicum*. *Gene* 145, 69–73.
