# Supplementary material for: The ColRS-Regulated Membrane Protein Gene XAC1347 Is Involved in Copper Homeostasis and hrp Gene Expression in Xanthomonas citri subsp. citri
Source: Front Microbiol. 2018 Jun 11;9:1171. doi: 10.3389/fmicb.2018.01171 (PMC6004745; doi:10.3389/fmicb.2018.01171)
Supplement: TABLE S2 — Primers for molecular cloning in this study. [file Table_2.DOCX]

Table S2. Primers for molecular cloning in this study

| **Primer pair** | **Sequence(5’-3’)** | **Cutting sites** | **Description or purpose** |
| --- | --- | --- | --- |
| 1347.1.F/1347.1.R | TGCTCTAGACGCGTTTGCCGACGAAATCGT  GCCGAGCTCTGTTTCAATCCTCGTCGTTAGATTA | *Xba*I-*Sac*I | A 720-bp DNA fragment upstream of *XAC1347* gene |
| 1347.2.F/1347.2.R | ATCGAGCTCTCACTTCTTGACTCCAAGCGA  ACGCTGCAGTCGTGCTTGTCGGCATTCTGTT | *Sac*I-*Pst*I | A 527-bp DNA fragment downstream of *XAC1347* gene |
| MColR.F/MColR.R | CGGAATTCTCAACCTGCCTGGCATGGA  CCCAAGCTTGCAGTTCTTCGCCCCACA | *Eco*RI-*Hin*dIII | A 399-bp DNA fragment of the partial sequence of *ColR* gene |
| C1347.F/C1347.R | TGCTCTAGACCATTGCTATCGAATTGATTTAATT  GCTCTAGAGGGAGACAGGCGCAATCTTGCAGGT' | *Xba*I-*Xba*I | A 631-bp DNA fragment containing *XAC1347* gene with its promoter |
| CColR.F/CColR.R | TCCTCGAGATCGGCCGGCTAACGGTT  TCAAGCTTCAGGCATCGGGCGAGGCG | *Xho*I-*Hin*dIII | A 1375-bp DNA fragment containing *ColR* gene with its promoter |
| 1347.G.F/1347.G.R | CCCAAGCTTATGACCATTAACAAGCTGCTGATCG  CGCGGATCCCTTCTTGGCTTCTTCTGCGGTGTCC | *Hin*dIII-*Bam*HI | A 342-bp full length of *XAC1347* fused in pGDG |
| 1347.P.F/1347.P.R | TGCTGCAGGCAATCACCGTGCTAATG  TCGGATCCTGTTTCAATCCTCGTCGTTAG | *Pst*I-*Bam*HI | A 542-bp XAC1347 promoter region |
| 1347.S. F/1347.S.R | CGTCTAGAATGACCATTAACAAGCTGCTGATCG  TGCGAGCTCTTACAAGTCCTCTTCAGAAATGAGCTTTTGCTCCTTCTTGGCTTCTTCTGCGGTGTCC | *Xba*I-*Sac*I | A 670-bp DNA fragment inserted into pBBR1MCS-5 for expressing C-Myc tagged XAC1347 at C-terminal |

The 5′ end of each primer contains a restriction enzyme site for cloning into the expression plasmids
