# Supplementary material for: The ColRS-Regulated Membrane Protein Gene XAC1347 Is Involved in Copper Homeostasis and hrp Gene Expression in Xanthomonas citri subsp. citri
Source: Front Microbiol. 2018 Jun 11;9:1171. doi: 10.3389/fmicb.2018.01171 (PMC6004745; doi:10.3389/fmicb.2018.01171)
Supplement: TABLE S3 — Primers for real time PCR analysis in this study. [file Table_3.DOCX]

Table S3. Primers for real time PCR analysis in this study

| **Gene products** | **Sequence(5’-3’)** | | **Product Sizes** |
| --- | --- | --- | --- |
|  | **Forward primer** | **Reverse primer** |  |
| *XAC1347* | ACGCCGCTCAGGCTGCTGCCAACAC | AGCCTGGTCAGCAGCAGCGTCGGTC | 107 bp |
| *hrcV* | TGGTCAACATCCTGGCCGGCAT | AGTGAGGCGATCTGCGACACCATG | 123 bp |
| *hrpF* | ACCGGATCTGAAGAAGGCATTGACG | CTTTGATCTTGCCGCCGCACTTG | 101 bp |
| *hrpB1* | GCGAACAGGCAGCAGGCGTACAA | TGGACACGTTCGATGCATGGATTTC | 133 bp |
| *hrpD6* | GGATCCTTCTGGCGAGCGGCTGC | ACGGCATTGAAGTCGTTGCGTGAGG | 120 bp |
| *gyrA* | TGGCCTCAAGCCTGTGCACCGG | GACGATACGCGCCGACTTGAAG | 100 bp |
| *ColS* | AAGGTGATGCGGCCGGTCT | AGTAGTCGTCCAGCGCTTCG | 138 bp |
| *ColR* | GCAAGCAGACCCCGGTGTT | TTGATCAGATAGTCGTCGGCG | 100bp |
